# Sediment replenishment combined with an artificial flood improves river habitats downstream of a dam

**DOI:** 10.1038/s41598-019-41575-6

**Published:** 2019-03-26

**Authors:** Severin Stähly, Mário J. Franca, Christopher T. Robinson, Anton J. Schleiss

**Affiliations:** 1 Laboratoire de Constructions Hydrauliques (LCH), École Polytechnique Fédérale de Lausanne (EPFL), CH-1015 Lausanne, Switzerland; 2 Water Science and Engineering Department, IHE Delft Institute for Water Education, 2611 AX Delft, The Netherlands; 3 Department of Hydraulic Engineering, Delft University of Technology, 2628 CD Delft, The Netherlands; 4 Department of Aquatic Ecology, Swiss Federal Institute of Aquatic Science and Technology (EAWAG), CH-8600 Dübendorf, Switzerland

**Keywords:** Hydrology, Restoration ecology, Riparian ecology, Ecosystem ecology

## Abstract

River reaches downstream dams where a constant residual flow discharge is imposed, often lack sediment supply and periodic inundation due to the absence of natural flood events. In this study, a two-year return flood was released from an upstream reservoir and combined with sediment replenishment to enhance instream habitat conditions downstream of Rossens hydropower dam on the Sarine River in western Switzerland. Sediment replenishment consisted of four sediment deposits distributed as alternate bars along the river banks, a solution which was previously tested in laboratory. The morphological evolution of the replenishment and of the downstream riverbed were surveyed including pre- and post-flood topography. A hydro-morphological index to evaluate the quality of riverine habitats, based on the variability of flow depth and flow velocity in the analyzed reach, was investigated. The combination of the artificial flood with sediment replenishment proved to be a robust measure to supply a river with sediment and to enhance hydraulic habitat suitability.

## Introduction

Sediment dynamics, referring to the transport and diversity in the size of sediment, are an often neglected but essential linkage in the nexus between water-food-energy and ecosystems. Flow and sediment dynamics are two interlinked key abiotic drivers in riverine ecosystems^1,2^ that host a large variety of habitats^3^. Water storage by river damming are vital infrastructures to guarantee food and energy production^4^, however these have a severe impact on both abiotic drivers since they regulate the flow and interrupt the longitudinal conveyance of sediment along the valley^5,6^, in particular in long reservoirs where the sediment continuum is often completely interrupted^6,7^. Upstream of a dam, deposited sediments cause the loss of storage capacity in the reservoir^8^. On the other hand, a lack of sediment causes river incision downstream of the dam, streambank erosion, reduction of morphology diversity and loss of habitats and fluvial connectivity^9,10^. In extreme cases, downstream sediment depletion leads to a new grain composition of the river bed with potential modification of the mass exchange between surface and sub-surface waters. Different measures to mitigate the downstream lack of sediments are applied in practice such as flushing, sediment bypassing and artificial replenishment of sediments, although with limited results and viability. Including the sediment dynamics in catchment management contributes to socially and environmentally sustainable water, food, and energy security, contributing directly to Agenda 2030^11–13^ in several of its targets.

Flood pulses released intentionally by dam owners, typically through their bottom outlets, can be combined with sediment replenishment in downstream reaches to deal with the problem of sediment dynamics^14,15^. Such reservoir flushing events in the case of significant reservoir drawdown can have significant impact on water turbidity^16^ and fish in downstream reaches and in the reservoir itself; hence such operations need to be carefully planned and monitored^17,18^. An experimental two-year return flood, combined with sediment replenishment to the channel bed, was tested downstream of Rossens Dam on the river Sarine in Switzerland. Sediment replenishment, analogous with one tested in laboratory, consisted of four deposits alternately distributed along both banks of the river^19^. According to the laboratory study, this configuration provides, compared to a classical replenishment of one single deposit, periodic sediment clusters on the river bed, thereby enhancing habitat diversity in a reach. In the current study, this type of sediment replenishment was tested for the first time in prototype combined with the release of an artificial flood. The results were evaluated in terms of hydraulic habitat suitability enhancement quantified by the Hydro-Morphological Index of Diversity (HMID)^20,21^.

## Case study: The Sarine floodplain

**Figure 1:**
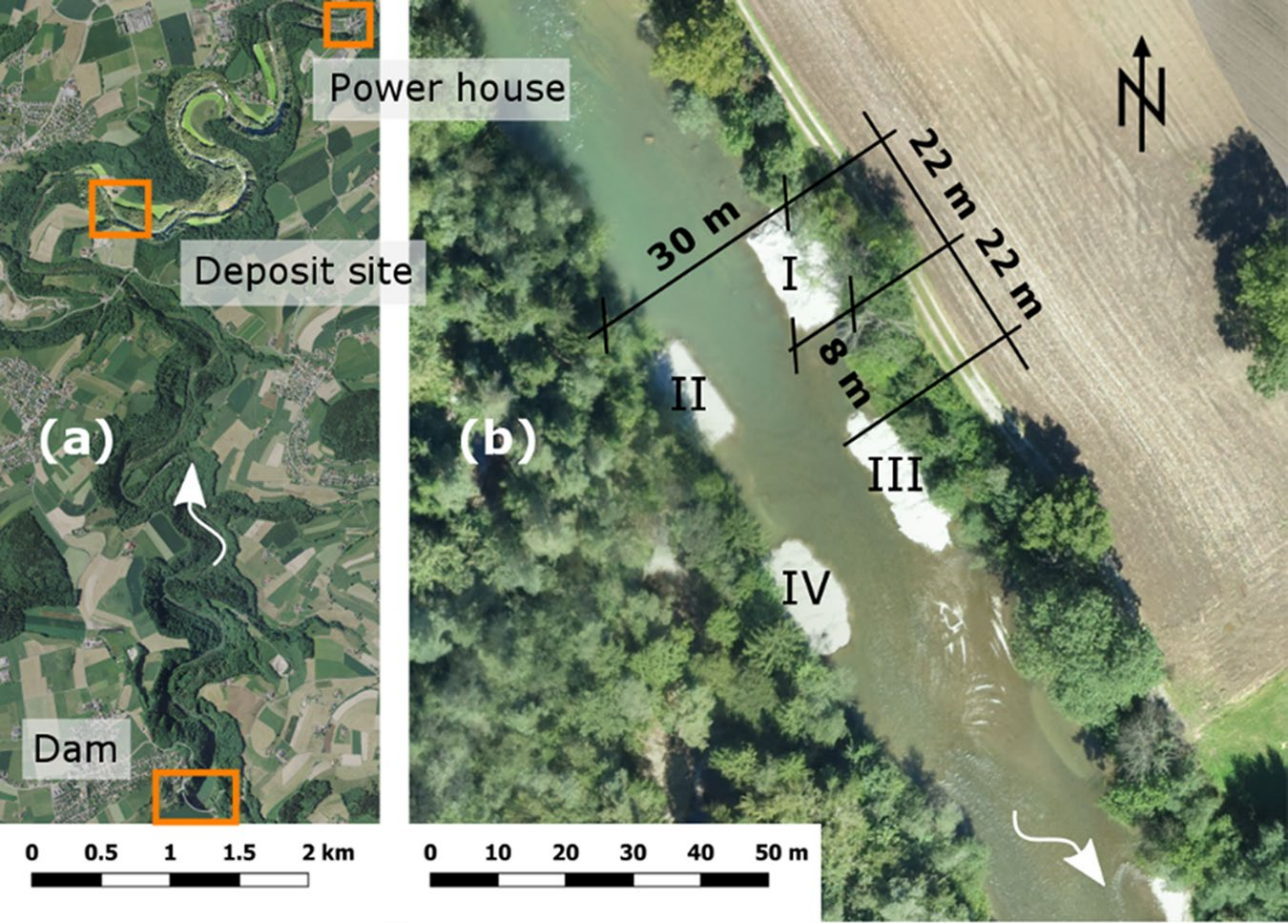
(**a**) Replenishment site and the locations of the dam and power house, background Geodata © swisstopo. (**b**) Configuration of the four sediment deposits in the Sarine River, background © Research unit Ecohydrology, ZHAW. The distance between the deposits was about a deposit length, the shift between the deposits on the left and the right bank is half a deposit length. The height of the deposits was ca. 1.5 m above the initial river-bed. The numbering refers to the name of the deposits from upstream to downstream: deposit I, II, III and IV.

The study took place at the Sarine River in the Canton Fribourg in western Switzerland. The Sarine originates at Sanetsch at 2252 m a.s.l. After 125 km it drains into the Aare, a tributary of the Rhine. Multiple reservoirs for hydropower production were built along the Sarine (Lac de Sénin, Lac du Vernex, Lac de Montbovon and Lac de la Gruyère), resulting in a regulated flow regime. The 83 m high Rossens arch dam forms the over 13-km long Lac de la Gruyère, which has a volume of 200 million m^3^. Being located in a pre-alpine valley, the lake is relatively long and sediment continuum is completely interrupted by the dam. Since construction in 1948, a residual discharge of 2.5–3.5 m^3^/s has been released by the Rossens Dam. This is the only discharge in the 13.4 km long reach with an average slope of 0.3% between the dam and the powerhouse in Hauterive (Fig. 1a). In Hauterive, up to 76 m^3^/s re-enter the Sarine during electricity production causing downstream hydropeaking.

This river segment of the Sarine has a meandering morphology. The floodplain lies in an naturally 100-m incised, wide, canyon revealing the cut-in of the river over thousands of years in the exposed steep rock walls at the outer bank of the river bends. However, in average, this river segment has a 1 m thick sediment layer above the bed rock. The Floodplain is surrounded by agricultural terrain and villages. Therefore, human-built structures in the floodplain are limited to some gravel roads, a handful of buildings and hiking paths. However, the absence of flood events and the interruption of sediment supply since the construction of the dam caused channel incision and disconnection of the floodplain. Vegetation colonized the previously wide gravel bars on the floodplain, mainly populated willows and hardwood.

The experiment was a collaborative research effort led by the cantonal authorities in collaboration with the dam owners (Groupe e) and engineering offices (Hydrique, Pronat) and with the participation of different research institutions (Zurich University of Applied Sciences, Swiss Federal Institute of Aquatic Science and Technology, University of Zurich and the École Polytechnique Fédérale de Lausanne). During the flood the investigations along the valley downstream of Rossens Dam had several scientific focus. The present research focused on the question how, through artificial replenishment of sediment, an increase of the variety of the bed channel morphology can be achieved. These morphology-driven objectives are linked to the more general goal of improving habitat diversity which is herein evaluated in terms of the index HMID.

## Results

### Sediment erosion and deposition after the passage of the artificial flood

**Figure 2:**
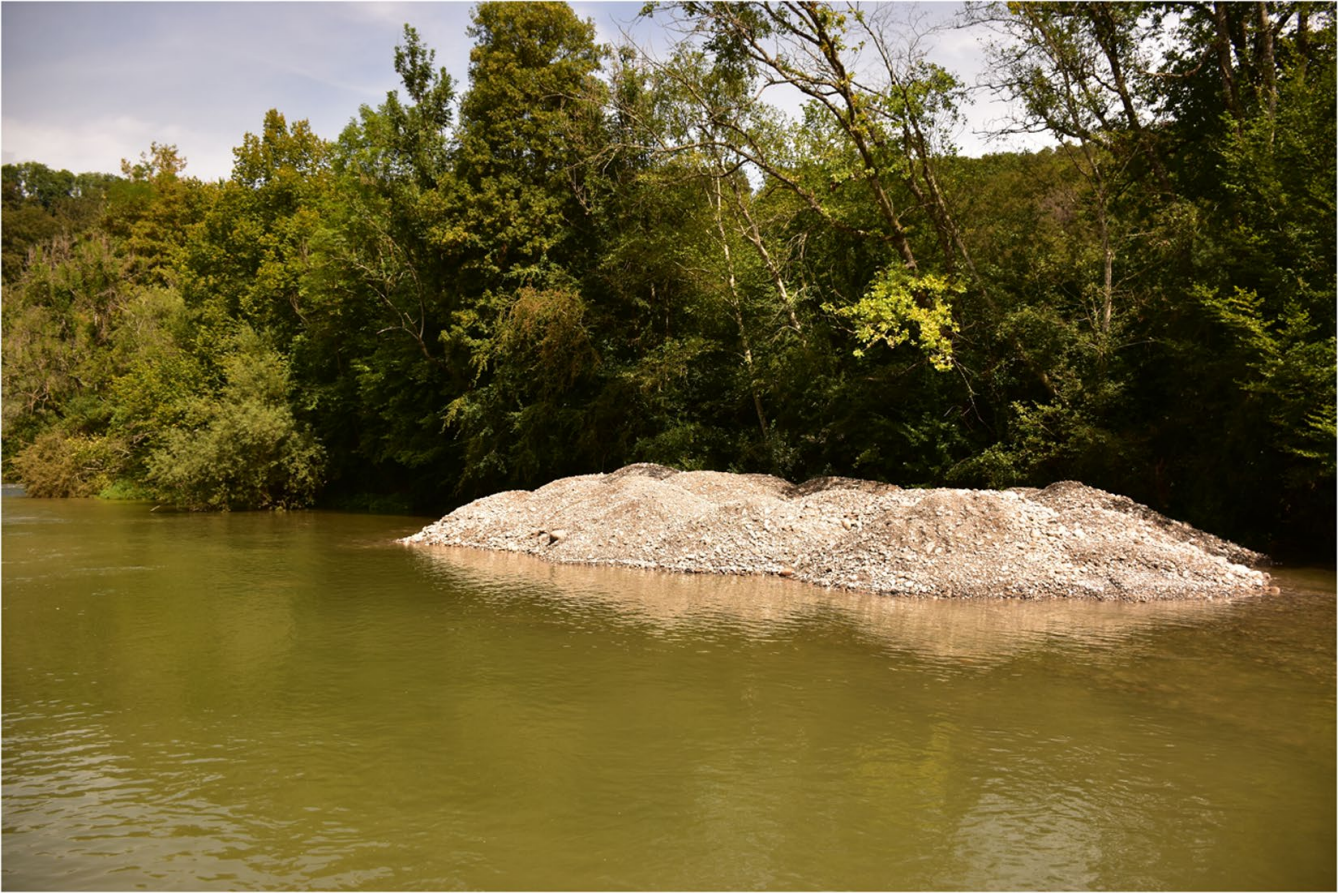
Deposit I in the river before the flood experiment. Its dimensions correspond to ca. 22 × 8 × 1.5m^3^ (length, width, height). The material was excavated from the adjacent riparian forest. Image © Elena Battisacco.

**Figure 3:**
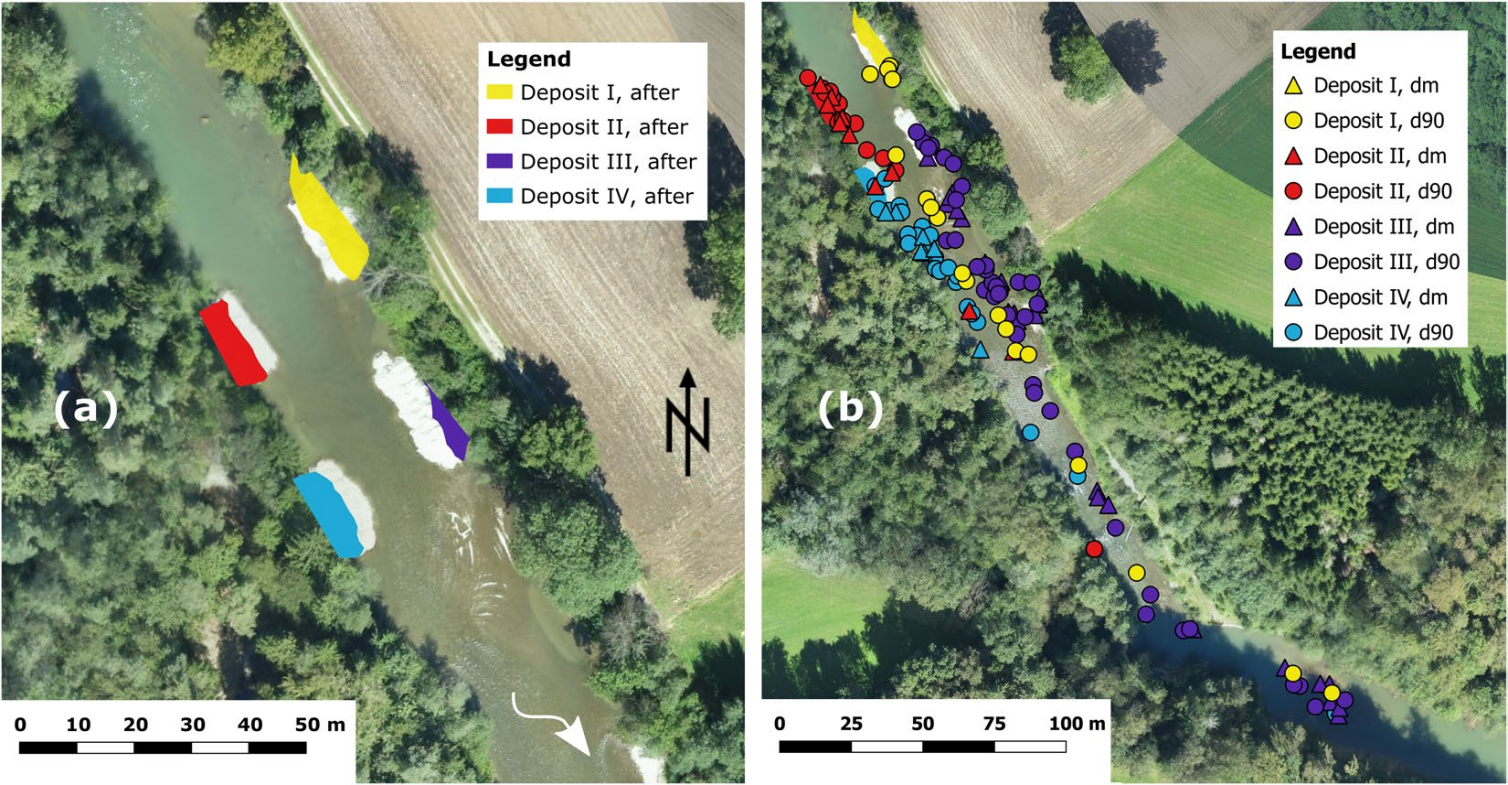
(**a**) The exposed dry surface of the four deposits before (air-image background © Research unit Ecohydrology, ZHAW) and after (colored areas) the artificial flood event. The residual flow discharge was 3.5 m^3^/s when the air-image was taken before the flood event and 2.5 m^3^/s when the dry surface was measured after the flood event. (**b**) RFID PIT tag equipped sediments detected after the flood event (background © Research unit Ecohydrology, ZHAW).

To follow the movement of the replenished material, 489 stones with a mean diameter of 5.7 cm (corresponding to *d*_*m*_) and 11.3 cm (corresponding to *d*_90_) were equipped with RFID PIT tags and added to each deposit (Fig. 2, see *Methods* for more details). The passage of the two-year flood was not strong enough to erode all the deposits (Fig. 3a). In deposits I, II and IV, sediment was mainly eroded laterally, whereas deposit III was fully eroded.

**Figure 4:**
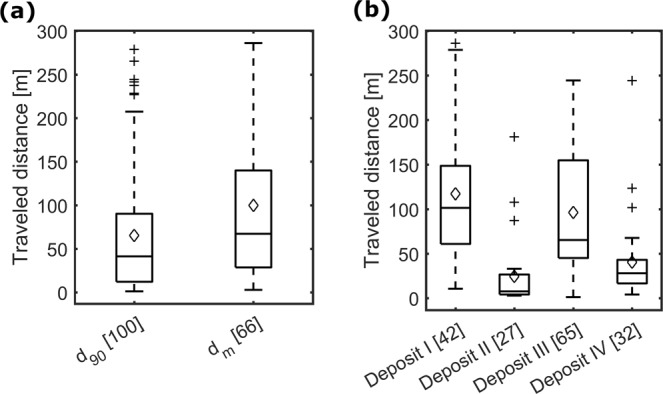
Traveled distances of the RFID PIT tagged sediments which were detected with the mobile antenna after the flood event sorted by (**a**) size and by (**b**) deposit of origin (see *Methods for following sediment transport* for details about the detection).

During surveys made after the artificial flood, 277 (57%) RFID PIT tagged sediments were recovered (Fig. 3b, see *Methods* for details about the detection). A total of 111 tags were detected on the remaining part of the deposits which was not eroded and 166 tagged sediments were eroded and transported downstream. The travelled distance depended mainly on the deposit of origin of the sediment and was always <286 m. On average, sediments with the size corresponding to the *d*_*m*_ travelled about 100 m and the sediments with the size corresponding to *d*_90_ about 65 m (Fig. 4a). However, their maximum transported distance is equivalent. Tagged stones from the deposit I and III were further transported than tags from deposit II and IV (Fig. 4b). Most of the detected tagged stones had deposit III as origin, followed by deposit I, IV and II. Tagged sediments from deposit I and III were all detected on the left side of the river, whereas the inverse was observed for sediments originating in deposits II and IV (Fig. 3b). The equipped tags settled in clusters, similar to the previous laboratory observations.

### Changes in hydraulic habitat suitability

Table 1 provides the results of the analysis concerning the change in habitat diversity using HMID (see methods for details about HMID). 200 measurement points of flow depth and flow velocity were measured on nine cross-sections in a 850 m long reach (more details in *Methods*). These data were then divided in three different sub-reaches: all nine cross-sections (corresponding to the 200 surveyed positions); three cross-sections lying in the area where the tagged sediments were detected (thus impacted by the replenishment of sediment, corresponding to 65 surveyed positions); and the remaining six cross-sections (corresponding to 135 surveyed positions). The results are always shown for before and after the artificial flood. In the impact area, where the tagged sediments were found, the HMID increased by 36%, whereas the HMID computed with the values sampled outside this impact area increased by 18%. The HMID outside of the impact zone was higher than in the impact zone, both before and after the flood event. The value of HMID outside of the impact zone corresponded to the lower limit of the classification of fully developed spatial dynamics (see Methods). This is confirmed by the presence of several geomorphology features such as pools, riffles and paleochannels.

**Table 1:** HMID values before and after the flood event and the sediment replenishment in the Sarine river.

			HMID_total_ (9 CS, 200 dps)		HMID_impact zone_ (3 CS, 65 dps)		HMID_rest zone_ (6 CS, 135 dps)	
			Before Flood Q = 3.5 [m^3^/s]	After Flood Q = 2.5 [m^3^/s]	Before Flood Q = 3.5 [m^3^/s]	After Flood Q = 2.5 [m^3^/s]	Before Flood Q = 3.5 [m^3^/s]	After Flood Q = 2.5 [m^3^/s]
μ_h_	[cm]		49.7	45.9	40.2	38.3	54.7	49.1
σ_h_	[cm]		29.4	31.4	18.2	22.6	32.8	34.0
μ_v_	[m/s]		0.43	0.39	0.45	0.42	0.42	0.38
σ_v_	[m/s]		0.34	0.33	0.28	0.31	0.37	0.35
HMID	[−]		**8.1**	**9.8**	**5.6**	**7.7**	**9**	**10.6**
HMID Variation			**+21%**		**+36%**		**+18%**	

*CS* stands for cross-sections, to the impact zone belong the cross-sections where the tagged sediments were found (CS 2–4), the rest zone corresponds to the sections where no sediment was found (CS 1, and 5–9, see Fig. 6). *dps* stands for data points representing the amount of flow depths and velocities used to calculated the corresponding HMID value.

## Discussion and Conclusion

The results clearly demonstrate that the release of an artificial flood combined with sediment replenishment of the riverbed significantly increased the morphological diversity of a river reach downstream of a storage dam, which in turn led to a significant amelioration in hydraulic habitat suitability. The results also point out that the sole release of the artificial flood in the valley downstream of Rossens Dam, to a certain extent, also enhanced conditions for hydraulic habitat suitability. Furthermore, a sole release of a flood pulse in the absence of sediment replenishment likely would lead to a wash-out of sediment in the bedrock-alluvial Sarine River and accelerate bed incision. A long-term field study is still missing as well as investigations with successive flood releases. However, all indicates that the HMID will be enhanced only in combination with a regularly supply of sediment during the artificial release of the flood. It is also important that sediment replenishment following this first positive restoration experience should be placed as close to the dam as possible to supply the entire river with sediment. The present results encourage further similar experiments and spark the discussion of further possibilities for the definition of environmental flows. This interdisciplinary approach, combining ecohydraulics, engineering and geomorphology, may be a crucial puzzle piece to define a proper sediment management in river reaches impacted by dams. The biological survey that was performed parallel to this study also revealed positive first effects of the flood in the Sarine^22^.

## Methods

### The artificial flood and the replenishment of sediments

**Figure 5:**
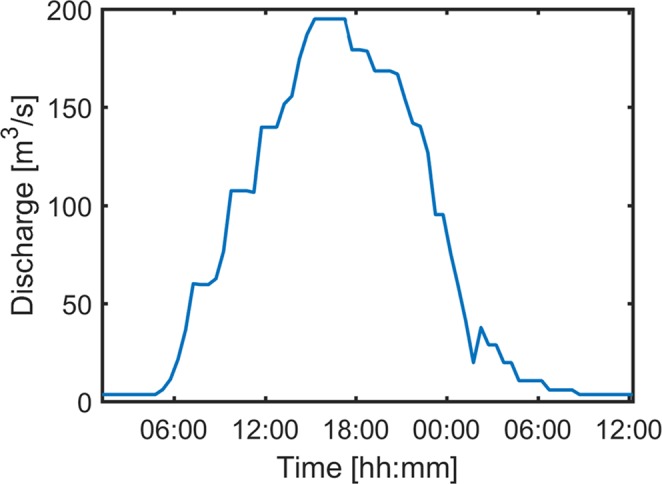
Hydrograph of the flood pulse. The peak discharge of this two year flood was limited by restrictions in the flooding perimeter. The whole flood lasted more than 24 hours starting from 14 September 2016 at 05h00 to 15 September 08h30.

A flood pulse with a peak discharge of 195 m^3^/s, corresponding to a return period of two years, was released from Rossens dam in September 2016. Due to the limitations of the hydrograph (polluted areas near the floodplain could not be flooded), a shape was chosen in which peak discharge was kept for two hours (Fig. 5).

In addition to the flood pulse, four sediment deposits were added to the river 9 km downstream of the dam (coordinates 46°45′29.21″N, 7°06′11.47″E; Fig. 1a) with a total volume of 1000 m^3^ (Fig. 2). The deposit lengths as well as the distance between deposits on the same river bank were slightly less than the river width (around 22 m, Fig. 1b). The mid-channel distance available for flow, in between deposits, was roughly half of the river width. The replenished material was excavated from the adjacent alluvial forest on the right bank of the river. The grain size distribution of the excavated material was determined analyzing 21 samples for pebble counts^23^ and photo sieving using BASEGRAIN^24^. The characteristic grain sizes were determined as *d*_50_ = 3.9 cm, *d*_65_ = 5.3 cm, *d*_*m*_ = 5.7 cm, *d*_84_ = 8.7 cm and *d*_90_ = 11.3 cm.

### Sediment tracking

Displacement of an individual sediment particle (stone) in a river can be tracked using radio-frequency identification technology (RFID)^25,26^. The passive integrated transponder (PIT) tags can be fixed in stones or artificial bodies with little effort and can be detected from a close distance. So far, RFID PIT tags proved to be a useful technology in wadable reaches with relatively low flow velocities (*v* < 1 m/s)^27^. Studies in different fluvial environments resulted in recovery rates above 85%^28,29^.

A total of 489 stones, divided into *d*_*m*_ and *d*_90_, were collected in the field, drilled, and the hole filled with a radio frequency identification passive integrated transponder tags (RFID PIT tag) of 32 mm or 23 mm length and silicon^30,31^. With the software S2Util, the tags were programmed with a unique identification number. After measuring the three axes and the weight of the tagged stones, they were distributed equally on the top, middle and bottom of the four deposits.

A mobile antenna consisted of a 150-cm long pole and an 80-cm diameter ring attached at the end, both made of plastic, was used. Inside the ring, a double loop of 4-mm^2^ multiple strand cable formed the antenna. The electro-magnetic components are portable. When detecting a PIT tag, the antenna made a noise and the tag ID was shown on the screen. Both antenna systems worked with a 12 V 7.0 Ah battery. The electromagnetic components, such as the PIT tags, the Tuning Board, HDX Ready and Control board were all provided by www.oregonrfid.com. The PIT tags work at a low frequency of 134.2 kHz, allowing the detection in submerged conditions.

With the help of the self-developed mobile RFID antenna, the location of the tagged sediments could be detected after the flood event^30,31^ and their locations were registered with a differential GPS (TOPCON HiPer Pro) providing an accuracy of a few centimeters. The locations of origin and settling for the tagged sediments could therefore be captured with a precision of less than one meter due to the ring-size.

### Hydro-Morphological Index of Diversity (HMID) to evaluate hydraulic habitat suitability

The changes in hydraulic habitat suitability after the combined artificial flood release and replenishment of sediments were quantified using the Hydro-Morphological Index of Diversity (HMID)^20,21^. The HMID is based on the following hypotheses: the structural diversity of a river reach can be characterized by the hydraulic measures of flow depth and velocity and their statistical parameters, which are in turn a surrogate for habitat suitability for the aquatic and semi-aquatic region of a river reach. The HMID takes into account the spatial distribution of flow depth and flow velocity, like other indexes to evaluate habitat and morphology heterogeneity^32,33^:

1 $$HMID=\prod _{i}{(1+C{V}_{i})}^{2}={(1+\frac{{\sigma }_{h}}{{\mu }_{h}})}^{2}\cdot {(1+\frac{{\sigma }_{v}}{{\mu }_{v}})}^{2}$$

where *CV* = coefficient of variation of variable *i*; *μ*_*h*_ and *μ*_*v*_ = mean value of flow depth (*h*) and velocity (*v*), respectively; and *σ*_*h*_ and *σ*_*v*_ are the corresponding standard deviations. Generally, HMID-values between 1 and 15 are obtained depending on the river morphology. A river reach can be categorized as^20,21^:

- 1 < HMID < 5: Channelized/heavily altered
- 5 < HMID < 9: Limited variability
- HMID > 9: Fully developed spatial dynamics

**Figure 6:**
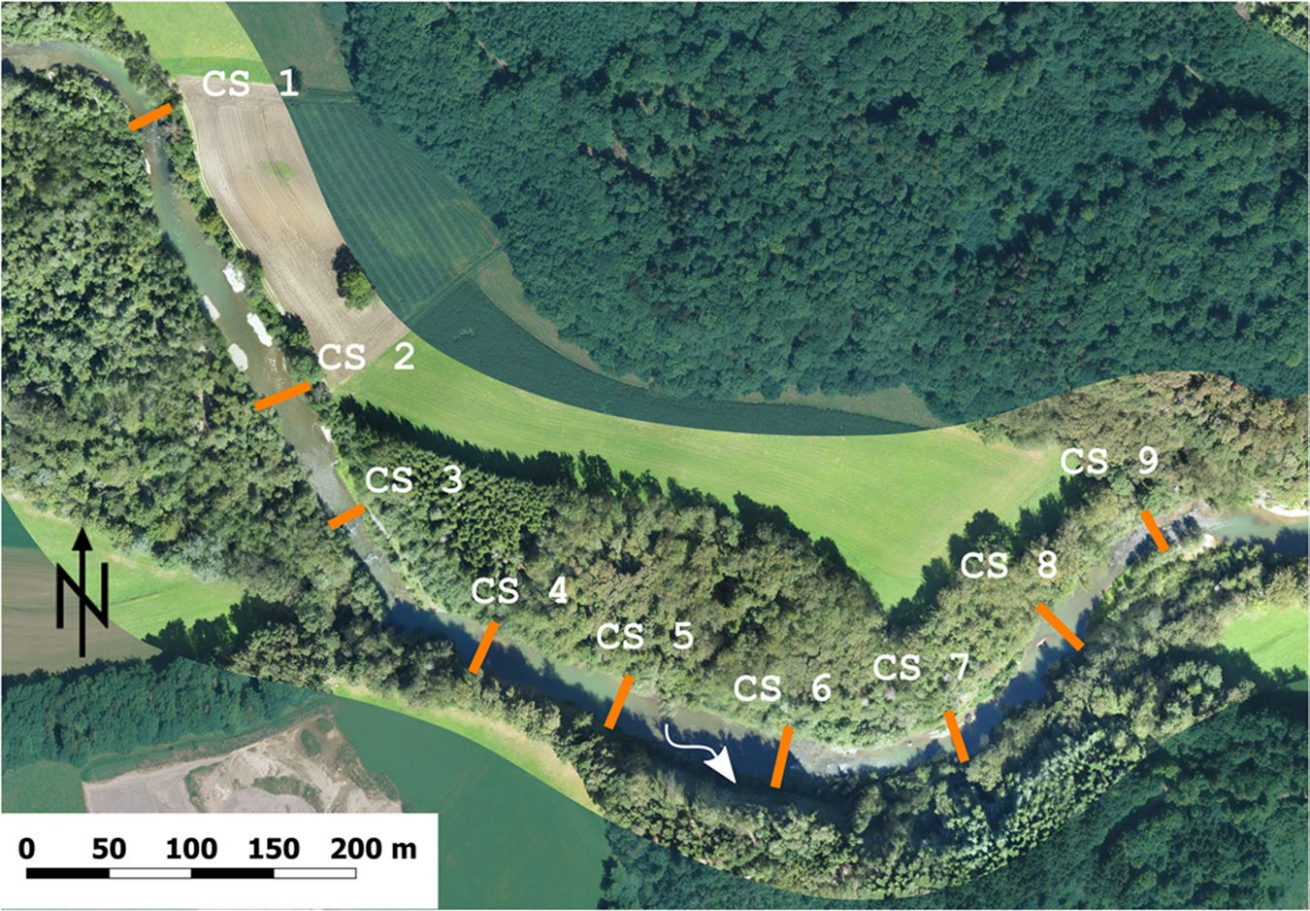
Nine cross-sections (CS) where flow depths and velocities were measured. CS 2–4 correspond to the HMID_impact zone_, CS 1 and 5–9 to the HMID_rest zone_ (see Table 1). Background © Research unit Ecohydrology, ZHAW.

A 850 m reach was analysed. Measurements of flow depth and flow velocity were made at the exact same locations before and after the flood event, across the river at nine different equally-spaced cross-sections with a distance of about 95 m (Fig. 6). A total of 200 velocity and water depth measurements were taken in the reach, before and after the flood event. The measurement points were equally distributed over the cross sections with a 1 m spacing. Instantaneous flow velocities were measured with a Handheld-ADV (FlowTracker manufactured by SonTek) at 60% of the flow depth. Flow velocities were averaged over a period of 45 s, with a measurement frequency of 1 Hz, which proved to provide a robust mean value. Flow depths were measured with the scale on the vertical bars of the FlowTracker equipment. The location of the sampling points was recorded with a differential GPS. An analysis of statistical convergence revealed that the results obtained with 200 points surveyed in the 850 m in the Sarine river are sufficient. Detailed information concerning sampling and data sufficiency can be found in literature^34^.

## Data Availability

The datasets analysed during the current study are available from the corresponding author on reasonable request.
